# Medical and Philosophical Intelligence

**Published:** 1816-12

**Authors:** 


### MEDICAL AND PHILOSOPHICAL INTELLIGENCE.

*To the Editors of the London Medical and Physical Journal.*

GENTLEMEN,

I SUSPECT that the *minim*, and the *drop*, are not unfrequently confounded together, as synonymous.—If I mistake not, such must be the case in Dr. Thomas's fifth edition, 1816, of his "Modern Practice of Physic," which, considered in a general point of view, is unquestionably a very excellent and useful work. —At page 282, for instance, we find, in catarrh, a draught to be taken at bed-time, containing 40 minims (which, duly appreciated, is equal to 80 drops) of tincture of opium!—and the same kind of error seems to have pervaded the whole work.

In order to prevent the possibility of a mistake in a matter of such importance, it might be proper in every author to guard against an error of this kind, by stating that the minim is the sixtieth part of a drachm, in measure; and that two drops of any tincture, or spirituous liquid, ordinarily speaking, are equal to one minim; taking care, if he use the minim character, that it be applied according to these rules of proportion.

Should it be judged proper, in a future edition of the above work, to attend to these observations, Dr. Thomas might, in the quaint phraseology of a well-known favourite dramatic character, reply to Mr. Woodham, to whom he is indebted for these remarks, "Thank you, good sir,—I owe you one." R. WALKER.

---

The *Harveian Oration* this year fell to the lot of Dr. HAWORTH, whose classical acquirements stand at least as high as any gentleman's in the College; nor were his hearers at all disappointed. —We heard nothing of any measures yet taken to remove from the present inconvenient situation, almost rendered ridiculous by the recollection of Mr. Foote's Dr. Last.—It is remarkable that there has been no alteration of any kind in the list of fellows since the last year, excepting that Dr. G. D. Yeates is removed thither from the list of candidates. The list of licentiates is increased by nine fresh names, and has lost only two by death, Drs. Lettsom and Khron, the oldest on the list, excepting Sequeira, who has since died at a very advanced age. This intelligence may be more agreeable able to the aged practitioners than to those who are expected to succeed them. Dr. Fitton's name now appears among the candidates for a fellowship. That list is considerably increased, and a new order of "Inceptor Candidates" created, consisting of eleven. The mortality has been greatest in the shortest lists. The licentiates in midwifery have lost Drs. Denman and Squires, both full of years. The extra-licentiates Drs. Binns, Silver, and Coulthurst, we believe of a similar description.

---

*Small-pox more than Twenty Years after the casual Disease.*—The Rev. Mr. Coleridge, Minister of St. Andrews, Holborn, has caught the small-pox, by visiting a family under that complaint, in the course of his ministerial duties. He had the small-pox when a child in arms; and, though since several times exposed to the disease, has remained uninfected till now. The Rev. Gentleman has kindly promised us a more minute account of his present and former situation.

---

*Oxalic Acid.*—The number of subjects lately poisoned by the oxalic acid renders a more than ordinary degree of caution necessary on this subject. The principal use, we understand, to which this chemical preparation is applied, is to clean boot-tops; and, as it is not expensive, it is not only bought and sold with little reserve, but left about to be used when wanted. We have reason to believe that a large quantity is necessary to produce any dangerous effect, but the dose is far from being ascertained. It has usually been mistaken for Epsom salts, when any accident has happened. The account of the appearances after death are differently described; and, we suspect in this, as in other cases, sufficient distinction has not always been made between the destruction of the stomach and the neighbouring parts by the supposed poison and by the gastric juice. On this we are engaged in a few experiments, the result of which shall be recorded in our next. Meanwhile we shall be thankful for any well-authenticated communications.

---

The Germans, and other continentalists, continue their cures of the venereal disease without mercury. When will they understand the writings of our English pathologist, and see the necessity of first ascertaining the disease? In a future Number we shall give the result of their remarks.

---

In two dissections, *post mortem*, in cases of hydrophobia, in the *Charité* at Berlin, the cerebrum was found overcharged with blood, and the œsophagus, trachea, pleura, palate, and epiglottis, superficially inflamed; and, in the first case, which had terminated fatally as early as twelve hours after the treatment was begun in that hospital, a smell of putrescency was perceived thirty-six hours *post mortem.*

*Observation*

*Observation of an Iliac Passion from a Rupture incarcerated in the Foramen ossium innominatorum.*—A delicate little woman, fifty-four years of age, was taken with colic pains, on the evening of the 30th of May, 1816, shortly after taking a draught of cold water when the body was heated. Her husband being a surgeon, imagined he should be able to alleviate these symptoms, as usual, with a few anodyne drops, instead of which the pains increased at short intervals, the patient complaining chiefly of the left side. The abdomen began to swell; nausea and vomiting gradually ensued, first of the injesta, and afterwards of a putrid black matter, of a very offensive odour, when syncope followed; till, on the 24th of June, her dissolution took place, from mere weakness, without any violent symptoms. Numerous enemeta came away just as they had been injected.

*Dissectio post mortem.*—The small intestines were found much distended and inflamed, and a small piece of the ilium was found incarcerated in that aperture of the foramen ovale which serves for the transmission of the nerves and blood-vessels.

Dr. Nuckel, of Limburgh, who communicates the above to Dr. von Wondelstadt, who attended as consulting physician, observes the slow progress of all the symptoms of this disorder, assumed after the first week, as the most remarkable in the case, the patient lying quiet, and almost without any febrile motion, and having even a desire for food, when not tormented by the nausea, vomiting, or pains, which, most probably, were only caused by the great distension and antiperistaltic motion of the intestines, taking its rise from the point of the stoppage. Equally remarkable were the intestinal windings, which were plainly visible through the thin abdominal integuments of the emaciated patient, and which might be observed like moving cords. They gave so striking a proof of the muscular power of the intestines, that the patient herself called them *a rummaging animal.* Regarding the cause of the disorder, it appears very probable, that the rupture had already taken place in the month of March 1814, when the patient, after slipping out on a glazed frost, was confined to her bed for seven weeks, with dreadful pains in the left groin; and that the incarceration was only induced by the inflammation, as a consequence of the cold draught while the body was heated. The failure of the remedies, and the pointing out of the painful place, indeed, left no doubt of the mechanical impediment of that side, yet the nearer determination remained undiscovered till after the dissection, which, indeed, could have availed but little, considering the difficulty of the operation, which, in this case, would have been the only remedy that might have been of any service. Dr. Wendelstadt observes, incarcerated hernia in the circumference of the musculi obturatorii foraminis ovalis ossium innominatorum pelvis, are phenomena of which B. Bell and A. G. Richter make mention, but whose existence other practitioners have doubted. This fact, however, decides unequivocally in favour of the learned gentlemen just named.

A long

A long communication from Germany, *on the Virtues of the Meloe Proscarabæus* (May-bug) *in Hydrophobia*, concludes thus:

Dr. VON EMBDEN'S *Remarks on the above.*—The power of this insect to operate on the urine and perspiration, indeed, being seated in all its parts, but more particularly in the wings, and, above all, in a yellow clammy juice contained in the vesicles next to the intestine, it appears not at all unlikely that the excrements of the meloe, being impregnated with that juice, shew greater efficacy than the other parts of it. The insect also forms an ingredient of an electuary which was sold, by a Silesian peasant, as a specific in canine madness; and which was bought by the King of Prussia, in 1777, and ordered to be used by the physicians throughout all his dominions; and, as it proved to be of great service, it was also adopted in other countries, with the omission only of the lead, which entered the composition, increasing the danger of its exhibition, without doing any good against the disorder. This electuary was prepared in the following manner:—Twenty-four May-bugs smothered in honey, together with the honey that adhered to them; two ounces of theraica; two drachms of ebony; one drachm of Virginian snake-root; and twenty grains of the mucus sorbi salviæ; were mixed with as much of the honey in which the insects had been lying as was sufficient to form an electuary. The dose for adults was ʒifs to ʒij (a proportionate smaller one to females and younger subjects). The patient was to eat nothing for twenty-four hours; and, for twelve hours, to drink only an infusion of elder flowers. When, by this method, he had got into a thorough sweat in the bed, his shirt and the bed-clothes were to be washed, and dried in the air, or entirely removed and destroyed. At the same time, the wound was to be washed; and kept open and moist with the oil of scorpions or May-bugs. Drs. Beireis and Dehre have cured hydrophobia by giving one-eighth to one-half of this insect for a dose, and continuing its use till bloody urine was produced, combining the remedy with scarifications, and daily applications of mercurial ointment, or pulvis cantharidum, to the wound; and Degner and Vogel also confirm the efficacy of this insect in counteracting the deleterious effects of the bite of rabious dogs.

---

From a correspondent, not of the faculty, we have the following hint: we presume it refers to the high operation, as it is well known lithotomy was practised before the days of Hippocrates.

*The first Operation for the Stone.*—In the month of January 1474, the physicians and surgeons of Paris represented to Louis XI. that several persons of consideration were afflicted with the stone, colic, iliac passion, and pain in the side; and that it would be highly desirable to examine the seat of the disorder, which could not be done so well as upon a living subject:—they therefore prayed that he would command to be delivered to them one Franc-Archer, who was condemned to be hanged for robbery, and who had been often grievously afflicted with these disorders.

Their

Their request was granted, and the operation was performed publicly in the church of St. Severin. After the examination was made, they replaced the bowels, stitched up the wound, and, by the king's order, great care was taken of the patient, who was completely cured in a fortnight. His crimes were pardoned, without any cost,* and he even had money given to him in the bargain.

---

At a time when the London College of Physicians has thought proper to decline the examination of male practitioners in the obstetric practice, it may not be amiss to show how attentive the Parisian physicians are, in whatever relates to the healing art.

*Les gardes malades*, or sick nurses, is a regular profession in France, and it were much to be wished that similar regulations were established in England. It is forbidden, under a severe penalty, that any person practise the profession of *gardes malades* without having previously undergone an examination, and obtained a certificate of competency from the College of Physicians. Their qualifications are, a kind and affectionate behaviour, a cheerful disposition, patience of fatigue, and readiness in the preparation of every thing, by way of diet, for the patient. They are sworn to administer the medicines faithfully, agreeably to the prescription of the physician, carefully to observe his orders in every particular, and to detail to him, at each visit, every symptom or occurrence that had taken place during his absence. To the ability, care, and vigilance of these humble auxiliaries, the French physicians are indebted, in some measure, for their high reputation; and it cannot be doubted that many valuable lives are thereby preserved to their families and to society.

The French *gardes malades* are subject to be suspended, and even disqualified, if in any case it can be proved that they have been wanting in any of the requisites, or have omitted to follow the orders of the physician; but this is a case that seldom happens. They are always gay and cheerful, and, instead of the downcast gloomy looks of affected sympathy, are lively, deceiving time by a fund of anecdotes of persons whom they had attended, *and who had recovered, though far worse than the present patient.* To those who have considered the action of the moral upon the physical powers, or, more plainly, the action of the mind upon the body, it will be evident, at first glance, how beneficial and important must be the effects produced on the pa-

---

* This alludes to a custom subsisting at the present day in France, where a criminal conviction carries costs, like a verdict in a civil court. Thus, one Firmin Bidant, who was guillotined for robbery on the 8th of May last, at Paris, was condemned to be beheaded, and to pay fifteen hundred francs, the costs of the proceedings. What renders such a circumstance occasionally ridiculous, is, that the sentence always contains such a clause, even though the wretch has not a rag to his back.

tient by such a mode of treatment, and how striking the contrast between it and the conduct of sick nurses in England, who, by affected concern, banish hope, the best prop of life; and, if the patient feel a nausea at taking medicines which might arrest the fleeting spark, will substitute for the prescriptions of an able physician some simple beverage, or, what is worse, indulge the patient's appetite in providing food highly improper for him. Let but the nurse's closet be examined after the decease of a patient, and it will generally be found that not one-fourth of the medicines prescribed have been administered: thus the life of many a valuable member of society, of many an affectionate parent and darling child, has been sacrificed to the ignorance and perversity of a foolish and obstinate sick nurse.

The nurse's task is more humble, but it is often as important: she is a *necessary* auxiliary; she ought to be to the physician what the soldier is to his officer, obedient to the word of command, and strictly execute (without presuming to judge of the propriety or expediency of the measure) his orders with the utmost minuteness and fidelity. This is an important subject, not only in its direct effects on the health of the patient, but also relatively on the character and reputation of the medical attendant; and we shall accordingly call the attention of the faculty to it in a future Number, in order to establish some legislative regulations on the subject.

---

*Employment of House-leek in Cases of Epilepsy;* by Dr. PETERS, of Anclam.—The epilepsy is one of the most afflicting disorders, and on which medicine has hitherto exercised but a very imperfect empire. Dr. Peters declares he has cured five patients by the administration of house-leek, dried in an oven, and pulverised. He says he received the remedy from his father, who had applied it with success.

Case I.—A woman, aged thirty years, had been subject to epilepsy for fifteen years, from a fright during pregnancy. The attacks returned irregularly, after a fit of anger or violent grief. She took, at first, twenty grains of houseleek, in an equal quantity of sugar, morning and evening. In the course of six weeks, she had only one slight attack, occasioned by a sudden emotion. She had then taken an ounce of house-leek. She continued the medicine, and took one ounce more, by which she was so entirely relieved that in the course of twelve months she had not the slightest return of the disorder.

Case II.—A widow, aged 40, had been subject to attacks of the epilepsy from the age of thirty, which took place at irregular periods, twenty or thirty times in the course of the year. Having taken ten grains of pulverized houseleek twice a-day during four months, she suffered no attack. After taking one ounce and a half, she had two slight attacks, which Dr. Peters believes to be the last.

Case III.—A boy, aged 10 years, epileptic from the age of seven. Dr. Peters administered eight grains twice a-day: after having taken half an ounce, her father wrote to Dr. P. to state that the attacks were becoming slight, and that the patient did not, as heretofore, become insensible.

Case IV.—A girl of 15, who had menstruated from the age of 14, experienced attacks of epilepsy generally at the period of menstruation. She took ten grains of pulverized houseleek twice a day; and was cured after taking one ounce and a half. At the time of noting this case, she still continued to take the remedy.

Case V.—This was a boy, aged 10, who had been affected several years with St. Vitus's dance. Having taken half an ounce of houseleek, in doses of eight grains, morning and evening, he felt considerably better, when he discontinued the medicine. Dr. P. is of opinion that if it had been continued for some time after the cessation of the disorder, a radical cure would certainly be the result.

---

*A new French Pharmacopœia.*—By an ordonnance of the King, bearing date the 8th August, 1816, the Codex Medicamentarius seu Pharmacopœia Gallica, ordered to be prepared by Napoleon in 1803, is forthwith to be printed; and, within the date of six months from its publication, every apothecary is bound to procure a copy, and always to prepare his medicines strictly according to the formula, under a penalty of 500 francs. Every copy is to be stamped, 1st, with the seal of the Faculty of Paris; 2d, with the signature of the president of the Faculty of Medicine at Paris; and 3d, with the bookseller's name. Every copy not so stamped and signed to be considered counterfeit, and the seller prosecuted by the King's attorney-general. It is a singular circumstance that there has been no French official Pharmacopœia published since the year 1748!

---

*Experiments on the Venom of the Viper;* by M. Mangili, Professor of Natural History in the University of Pavia.

The beautiful experiments of Redi and Fontana on the venom of the viper, have proved, that the danger arising from its bite, is always in proportion, on the one hand, to its own size, and that of the animal bitten; and, on the other, to the quantity of venom introduced into the wound, which depends on the period elapsed since the viper had last stung an animal, and the degree of anger excited in it; for, when enraged, the glands which secrete the venom, are more active, and furnish a greater quantity, and, perhaps, also render it more powerful.

These experiments have also proved, that the venom of even the largest vipers of Italy are not sufficient, under ordinary circumstances, to kill a man, and that the inflammation arising from the wound, would disappear of itself, in a few days, without leaving any disagreeable consequences.

The results directly obtained by these learned gentlemen, have since been frequently corroborated by events occurring in every part of Europe. I do not, it will be perceived, take into the account, account, all the tales collected on this subject by credulity, or of accidents by fear—for fear itself frequently commits homicide.

The researches of Professor Mangili perfectly agree with the facts stated by his predecessors; we shall also find in them some particulars, which prove how unfounded are many of the medical theories with which Italy is infected.

M. Mangili, in the course of his demonstrations, having handled a viper carelessly that he had irritated much, it bit him severely under the nail of the left thumb. He instantly pressed all the blood he could from it, and extracted more by suction; and, not to interrupt his lesson, he drank half a bottle of excellent Malaga, which enabled him to continue his experiments. At the conclusion, finding himself a little weak and faint, he drank twice, in a glass of water, from fifteen to twenty drops of ammonia (*alkali volatil fluor*), and took a walk in the country. In the evening, finding the thumb swelled and painful, he bound it up with a bandage of theriaca, and even took a dose of that remedy, which procured him ease from pain, and enabled him to sleep. An emollient cataplasm, in twenty-four hours, entirely removed the swelling and pain. M. Mangili adds, that, for several days, he had a weak pulse, and was obliged to have recourse to nutritive stimulants to recover the tone of his nerves.

Other experiments, by this professor, have proved still more conclusively, that Nature alone is sufficient to triumph over the bite of the viper, when the animal bitten is of the proper size. He caused a large dog to be bitten in the nose by a strong viper. The dog was at first furious, but soon became extremely mild,—then stupid and *engourde*. After appearing very low-spirited, he threw himself on the ground in agony; he vomited several times, and remained five days without eating or drinking. The sixth day, he ate, with an appetite, a little meat, and drank some cold milk. He was able to walk about on the seventh; and, on the eighth, he was well.

A very large strong rat was bitten by a viper with which it was confined; it became stupified in about twenty minutes, fell motionless, and died. Another, on the contrary, attacked by more serious symptoms, having been exposed to the free air for about two hours, recovered by degrees.

The opinion of several celebrated physicians of Italy being, that opium and musk stimulate in the same manner as ammonia, and, that they are perfectly congenial in their stimulating action; M. Mangili made experiments to discover, whether these two substances would, like ammonia, destroy the action of the venom of the viper in the animal economy. He commenced, by collecting the venom of twenty large vipers, which he dried and reduced into very small fragments, as more convenient in the experiment he proposed to undertake.

With the venom thus prepared, he inoculated two chickens, and administered a grain of opium to each; one of them died in three hours; the second manifested signs of being poisoned after two

 half had elapsed; two grains more of opium were administered in generous wine. It seemed to reanimate it a little, yet, even four grains and a half more administered to it, could not, in any degree, destroy the virulent action of the poison.

A strong young chicken had given to it, six grains of opium, at an interval of two hours between each grain. The next day, six grains more were administered; and, an hour afterwards, a small quantity of the dried venom was introduced into the thigh. In three quarters of an hour, it exhibited symptoms of stupor and fainting, interrupted by convulsive motions. It was in agony two hours and died.

The author then administered ten grains of opium, at once, to a strong hen, and twenty-four grains to another. They both died; the first, in three hours; the second, in four hours, after being infected with the venom.

Forty-eight grains of opium were administered to a strong turkey, which was bitten two hours afterwards by a viper. Signs of being poisoned were soon manifested; and it died in about three hours in a long but tranquil agony.

He made the same experiments with musk. Several fowls, to which he administered from two to twenty-five grains, died from the insertion of the venom in the thigh, half an hour, or three-quarters of an hour, afterwards.

Dr. Mangili concludes, from these various experiments, first, that ammonia, or liquid volatile alkali, is the sovereign remedy against the bite of the viper.

Secondly, That the vital forces alone, may, in some rare cases, triumph, without the aid of art, over the venom of the viper, when it is not in sufficient quantity to extinguish life!

That opium and musk, although congenial with ammonia in their stimulating properties, ought not to be preferred, by a prudent physician, to combat such dangerous accidents.

---

Dr. Merriman and Dr. Ley will re-commence their Course of Lectures on Midwifery and the Diseases of Women and Children, on Saturday, December 14th, at the Middlesex Hospital.

Dr. Davis, Physician to the Queen's Lying-in Hospital, and to the General and Central Lying-in Charities, &c. will commence a Course of Lectures on the Theory and Practice of Midwifery, and on the Diseases of Women and Children, on Thursday the 12th of December, at six o'clock in the evening, at Mr. Taunton's Theatre, 87, Hatton Garden.

Mr. Parkinson has announced a second edition of the Hospital Pupil, corrected and enlarged, and divided into two addresses. One of these, to parents and guardians, contains observations on the necessary previous education, and on the pecuniary resources of such as are intended for the practice of medicine and surgery; with suggestions as to an improved course of professional instruction. The other is addressed to the pupils themselves, on the order of their studies, &c. with hints on entering into practice, and on medical jurisprudence.

### METEOROLOGICAL REGISTER.

*From October the 26th, to November the 25th,* 1816.

Kept by C. BLUNT, Philosophical Instrument Maker, No. 38, Tavistock-Street, Covent-Garden.

| Moon. | Day. | Wind. | Barometrical Pressure. Max. | Min. | Mean. | Temperature. Max. | Min. | Mean. |
|---|---|---|---|---|---|---|---|---|
| | 26 | SE | 29·42 | 29·35 | 29 38 | 56 | 40 | 44·75 |
| ◐ | 27 | SE | 29·82 | 29 66 | 29·74 | 55 | 36 | 45·75 |
| | 28 | S | 29 90 | 29·89 | 29·90 | 57 | 36 | 46 25 |
| | 29 | S | 30·40 | 30 38 | 30·39 | 55 | 35 | 44·5 |
| | 30 | SW | 30·49 | 30·46 | 30·47 | 56 | 33 | 42·75 |
| | 31 | W | 30·37 | 30·22 | 30·29 | 55 | 34 | 42·5 |
| | 1 | S | 30 02 | 29 88 | 30·45 | 55 | 35 | 43· |
| | 2 | SE | 29 73 | 29·59 | 29 64 | 54 | 32 | 41·5 |
| | 3 | SE | 29·62 | 29·58 | 29 60 | 53 | 27 | 39·25 |
| | 4 | E | 29 57 | 29·56 | 29 57 | 53 | 30 | 38·5 |
| ○ | 5 | E | 29·52 | 29·50 | 29·51 | 55 | 35 | 43 5 |
| | 6 | E | 29·40 | 29·37 | 29 39 | 56 | 37 | 46 5 |
| | 7 | W | 29·30 | 29 28 | 29.29 | 56 | 32 | 43·75 |
| | 8 | W | 29 20 | 29·19 | 29·20 | 56 | 32 | 43·25 |
| | 9 | NW | 29·40 | 29·38 | 29·39 | 55 | 38 | 44 75 |
| | 10 | N | 29·72 | 29·70 | 29·71 | 53 | 40 | 46·25 |
| | 11 | NE | 29·50 | 29 48 | 29 49 | 55 | 42 | 48·5 |
| ◑ | 12 | NE | 30·38 | 30·20 | 30 34 | 55 | 41 | 46· |
| | 13 | E | 30 43 | 30·42 | 30·42 | 54 | 33 | 43 5 |
| | 14 | E | 30·45 | 30 43 | 30·44 | 52 | 31 | 42·25 |
| | 15 | SE | 30·43 | 30·28 | 30 35 | 50 | 29 | 38 75 |
| | 16 | E | 29 05 | 29·86 | 29·95 | 49 | 25 | 36 75 |
| | 17 | E | 29 07 | 29 94 | 30· | 49 | 23 | 36·5 |
| | 18 | E | 30·12 | 30 08 | 30·10 | 46 | 25 | 34· |
| ● | 19 | SE | 30 10 | 30· | 30·05 | 45 | 25 | 33·5 |
| | 20 | SE | 30·14 | 30·10 | 30·12 | 46 | 30 | 36· |
| | 21 | E | 30·20 | 30 16 | 30·18 | 46 | 30 | 37· |
| | 22 | E | 30·25 | 30·20 | 30·23 | 45 | 28 | 35·5 |
| | 23 | E | 30·32 | 30 22 | 30·27 | 46 | 28 | 35·25 |
| | 24 | NW | 30·21 | 30·19 | 30 20 | 46 | 28 | 34 75 |
| | 25 | NW | 30· | 29·90 | 29 95 | 40 | 34 | 37·80 |

RESULTS.

Mean barometrical pressure of the month 29·96

Maximum 30·49, wind at SW

Minimum 29·20, ——— W

Mean temperature of the month 38·97 deg.

Maximum 57, wind at S

Minimum 23, ——— E

*Scale exhibiting the prevailing Winds during the Month.*

| N | NE | E | SE | S | SW | W | NW |
|---|---|---|---|---|---|---|---|
| 1 | 2 | 11 | 7 | 3 | 1 | 3 | 3 |

| | Mean barometrical pressure. | Mean temperature. |
|---|---|---|
| From the first quarter on the 26th Oct. to the full moon on the 5th Nov. | 29·76 | 44·75 |
| ——— the full moon on the 5th, to the last quarter on the 12th | 29·79 | 42·40 |
| ——— the last quarter on the 12th, to the new moon on the 19th | 29·79 | 44· |
| ——— the new moon on the 19th, to the end of the period | 29·98 | 45·6 |

REPORT

### REPORT OF DISEASES.

The town is become particularly healthy, as is often the case after an epidemic of any kind. Sydenham usually remarked that seasons in which the constitution of the air was particularly free from any other epidemic, were always attended with small-pox, measles, and the other exanthemata. Such is very much the case at this time. How can we account for the existence of small-pox when the means of preservation are so easy? Surely some method might be discovered to render vaccination more general, if not universal. We were particularly led to these reflections by the following paragraphs, extracted from the same sheet of a daily newspaper.

"On the 20th of August last, a poor sailor was found lying in a street near Whitechapel, very ill with the confluent small-pox. A gentleman passing by put him into a hackney-coach, and paid the fare to the Small-pox Hospital, at Pancras: the coachman drove into the field at the front of the house, where he set down his fare, and left him. The resident surgeon went out to him, and, although he came without any recommendation, yet his case was so severe that he received him into the house. He was recovered of the small-pox, and also of a far more dangerous mortification; and, on the 7th inst. appeared to the Committee to express his thanks. Being entirely destitute, a small subscription was raised for him, and the Steward was ordered to supply him with some articles of apparel. He stated himself to have been paid off from the Berwick, of 74 guns, Capt. Brace, about three months since; that he caught the disorder at Plymouth, where he spent all he had received, and travelled on foot, during eight days, to London, in a very ill state, lodging in the open fields and under hedges at night. The poor man, after so long a residence and recovery at the hospital, could scarcely express his gratitude before his departure."

"On Saturday, an inquest was held at the House of Industry, Tewkesbury, before Henry Fowke, Esq. Coroner of that Borough, on the body of Richard Godsall, a labourer, belonging to the parish of Powick, in the county of Worcester, who died there the preceding evening, of that most dreadful malady the confluent small-pox.—From the testimony of the widow of this poor unfortunate and neglected man, it appeared, that he was taken ill at Badgworth, where he had been in employ a considerable time, on the Wednesday se'nnight preceding; and that, on the following Sunday, application was made in his behalf to the parish officers, assembled in the church-yard after divine service, from the behaviour of whom he was induced immediately to quit the place, and proceed on his way to his own home. From the advanced state of the disorder, however, he could only reach the neighbourhood of Churchdown that evening, where he and his wife lay in a desolate barn, without necessary sustenance, and without more clothes than his usual labouring dress. On the

 Monday,

Monday, he with great difficulty reached the Hamlet of Twigworth, in the parish of St. Mary-de-Lode, Gloucester, when he could proceed no farther, and applied to the overseer for relief, who put him into a hay-loft, where he lay all night among the hay and straw, without sheet or blanket, or covering of any kind, save his own clothes. The disorder had now made such a rapid progress in his constitution, that in the morning he exhibited a sad spectacle of human misery, totally blind, and so weak and emaciated that he was unable to stand. The overseer, notwithstanding, got him lifted into a cart, and ordered him to be conveyed through several intervening parishes to Tewkesbury, and there left. To save him from the jolting of the cart, his wife supported him in her arms the whole way; and, when they arrived at Tewkesbury, the wretched state of the poor sufferer exceeded all description. He was immediately conveyed to the House of Industry, where the best medical aid, and every solacing effort, were promptly resorted to; but all was too late to save him, and he languished in increasing affliction until Friday morning. The jury, after a minute investigation, returned a verdict—That the deceased, being violently ill of the small-pox at Twigworth, was removed from thence to Tewkesbury, on the 29th ult. and died of the said disease on the 1st instant, and that his death was greatly accelerated and hastened by such removal."

It has been sometimes suggested that the Small-pox Hospital was useless. We now begin to fear that the governors of that venerable establishment have been premature in alienating any part of their building; and trust that they have not done it without some reserve, which may enable them to resume it, if necessary.

Scarlatina is still general; and, during the severe weather, has been fatal in several instances. We have heard but little of measles. Peripneumony and other inflammatory diseases are still sporadic, with less violence, but requiring great attention.

